# ATF2-Induced Overexpression of lncRNA LINC00882, as a Novel Therapeutic Target, Accelerates Hepatocellular Carcinoma Progression *via* Sponging miR-214-3p to Upregulate CENPM

**DOI:** 10.3389/fonc.2021.714264

**Published:** 2021-08-27

**Authors:** Hua Ren, Zhi-cheng Wei, Yan-xia Sun, Chun-yan Qiu, Wen-jue Zhang, Wei Zhang, Tao Liu, Xu Che

**Affiliations:** ^1^Department of Radiation Oncology, National Cancer Center/National Clinical Research Center for Cancer/Cancer Hospital & Shenzhen Hospital, Chinese Academy of Medical Sciences and Peking Union Medical College, Shenzhen, China; ^2^Department of Hepatobiliary Surgery, National Cancer Center/National Clinical Research Center for Cancer/Cancer Hospital & Shenzhen Hospital, Chinese Academy of Medical Sciences and Peking Union Medical College, Shenzhen, China; ^3^Department of Etiology and Carcinogenesis, National Cancer Center/Cancer Hospital, Chinese Academy of Medical Sciences and Peking Union Medical College, Beijing, China; ^4^Department of Pancreatic and Gastric Surgery, National Cancer Center/National Clinical Research Center for Cancer/Cancer Hospital, Chinese Academy of Medical Sciences and Peking Union Medical College, Beijing, China; ^5^Department of Oncology Rehabilitation, Shenzhen Luohu People’s Hospital, Shenzhen, China

**Keywords:** lncRNA LINC00882, hepatocellular carcinoma, miR-214-3p, ATF2, metastasis, CENPM

## Abstract

**Background:**

Long intergenic non-protein coding RNA 882 (LINC00882) are abnormally expressed in several tumors. Our research aimed to uncover the functions and the potential mechanisms of LINC00882 in hepatocellular carcinoma (HCC) progression.

**Methods:**

RT-qPCR was applied to identify LINC00882 and miR-214-3p levels in HCC specimens and cells. Luciferase reporter was applied for the exploration of whether activating transcription factor 2 (ATF2) could bind to the promoter region of LINC00882. Cell proliferation, invasion, and migration were evaluated. *In vivo* tumor xenograft models were constructed to assess tumorigenicity. RT-PCR, Western blot and Luciferase reporter assays were conducted to examine the regulatory relationships among LINC00882, miR-214-3p and ATF2.

**Results:**

LINC00882 was markedly upregulated in HCC cells and clinical specimens. Additionally, ATF2 could bind directly to the LINC00882 promoter region and activate its transcription. Loss-of-function studies further demonstrated that LINC00882 knockdown inhibited proliferation, invasion, and migration of HCC cells. Mechanistically, LINC00882 adsorbed miR-214-3p, thus promoting the expressions of CENPM. Rescue assays demonstrated that functions of LINC00882 deficiency in HCC cells were reversed through suppressing miR-214-3p.

**Conclusion:**

Our group identified a novel regulatory axis of ATF2/LINC00882/miR-214-3p/CENPM, which may provide potential therapeutic targets for HCC.

## Introduction

Hepatocellular carcinoma (HCC) is one of the most common malignant cancers (1). It has been reported that there are 783,800 new cases diagnosed HCC worldwide each year (2). The main risk elements for HCC include hepatitis B virus, infection, cirrhosis, and accumulation of aflatoxins in the liver (3). Although more and more advancements have been achieved to develop life expectancy of HCC patients, postoperative metastasis and recurrence remain major disincentives for the improvements of clinical outcomes of HCC patients (4–6). Therefore, for personalized treatment and improvement of clinical efficacy, it is an urgent need to develop specific molecular biomarkers.

Long noncoding RNAs (lncRNAs) are RNAs with > 200 nucleotides in length without protein coding capacities (7). They are involved in almost all cellular and pathological processes, including cellular growth, apoptosis and stem cell maintenance (8, 9). Dysregulation of some lncRNAs has been verified in various tumors, such as lung tumor, gastric cancers, bladder cancers, and HCC (10, 11). Growing evidence indicates that lncRNAs act as tumor supporters or tumor suppressors by controlling cellular processes, such as differentiation, proliferation, invasion, and metastasis, highlighting their great potentials in tumor screening, prognosis, and therapies (12, 13). Although the involvements of lncRNAs in HCC initiation and progression have been studied in several studies such as lncRNA MIAT and lncRNA-PDPK2P, the potential exploration of lncRNA functions in the HCC contexts was located on their infancy (14–16). The novel mechanisms involved in lncRNA effects on HCC progression deserve in-depth explorations for the development of personalized treatments.

Long intergenic non-protein coding RNA 882 (LINC00882), located on 3q13.12, was a new cancer-related lncRNA (17). Several studies have reported its dysregulation in a few cancers, such as chromophobe renal cell carcinoma and hepatocellular carcinoma (18, 19). However, its specific function in HCC has not been investigated. In this study, we analyzed TCGA datasets, finding LINC00882 was highly expressed in HCC. Then, we further provided evidences of LINC00882 upregulation in HCC specimens from our patients. Furthermore, our group conducted functional assays to study the possible influence of LINC00882 dysregulation on tumor behaviors, and also studied the potential molecular mechanisms.

## Materials and Methods

### Clinical Specimens

A total of 6 pairs of tumor specimens and the matched non-tumor specimens were collected from National Cancer Center/National Clinical Research Center for Cancer/Cancer Hospital & Shenzhen Hospital. Chemotherapy or radiotherapy were not used for all patients before the operation. All patients gave informed consent and signed a written consent form. This research was ratified by the Ethics Review Committee of National Cancer Center/National Clinical Research Center for Cancer/Cancer Hospital & Shenzhen Hospital.

### Cell Lines and Cell Culture

The normal liver cells (HepaRG) and HCC cell lines (SMMC-7721, HepG2.2.15, HepG2 and Huh7) were purchased from ATCC(Manassas, VA, USA). HCC cells were cultured in a RPMI 1640 medium containing 10% fetal bovine serum (FBS) and HepaRG cells in keratinocyte-SFM (Thermo Fisher, Jijie Technology, Wuhan, China), together with 100 U/mL penicillin(Baomanbio, Pudong, Shanghai, China) and 100 μg/mL streptomycin(Yita Bio, Pinggu, Haidian, Biejing, China) with 5% CO_2_ at 37°C.

### Cell Transfection

Short-hairpin RNA oligos directed against LINC00882 (sh-LINC00882-1 and sh-LINC00882-2) were synthesized. Then, oligos were ligated into the shRNA vectors (JunHui Bio, Guangzhou, Guandong, China). To increase the expressions of ATF2 in HCC cells, the expressing plasmids for ATF2 were PCR-amplified and subcloned into the pcDNA3.1 vector, respectively (JunHui Bio, Guangzhou, Guandong, China). An empty pcDNA 3.1 served as a control. Ruibio Biology(Guangzhou, Guangdong, China) provided miRNA-214-3p mimics and miRNA-214-3p inhibitors. ATF2 small interfering RNA (si-ATF2-1 and si-ATF2-2) and the corresponding control RNA (si-NC) were purchased from Biomics Bio (Jiangsu, China) Co., Ltd. For cellular transfection, Lipofectamine 3000 (L3000015, Invitrogen, Ruidei Biology, Suzhou, Jiangsu, China) was applied based on the operating instructions.

### Quantitative Real-Time Polymerase Chain Reaction

Total RNAs in specimens and several cells were extracted by TRIzol reagent (Invitrogen, Beiyu Bio, Nanjing, China). Then, total RNAs were synthesized to cDNA by the use of PrimeScript RT reagent Kit (TaKaRa, Chengdu, Sichuan, China) based on the operating instructions. SYBR^®^ Premix Ex Taq ™ and StepOne Plus Real-time PCR System were applied to perform RT-PCR reactions. The primers were shown in **Table 1**. GAPDH or U6 served as an internal control. The relative expressions were calculated by the use of the ΔΔCt methods.

**Table 1:** The primers used in this study for RT-PCR.

Names	Sequences (5’-3’)
LINC00882: Forward	GCCGATACTTGACCTACGCA
LINC00882: Reverse	AGATGGCAGGTGCAATCACA
ATF2: Forward	AATTGAGGAGCCTTCTGTTGTAG
ATF2: Reverse	CATCACTGGTAGTAGACTCTGGG
miR-214-3p: Forward	GCACAGCAGGCACAGACA
miR-214-3p: Reverse	-CAGAGCAGGGTCAGCGGTA
CENPM: Forward	CGACCTGAACAGGGCTACC
CENPM: Reverse	ACGCACAGTCATCTTTGAGCA
GAPDH: Forward	GGAGCGAGATCCCTCCAAAAT
GAPDH: Reverse	GGCTGTTGTCATACTTCTCATGG
U6: Forward	CTCGCTTCGGCAGCACA
U6: Reverse	AACGCTTCACGAATTTGCGT

### Subcellular Fractionation

Nuclear and cytoplasmic separation was carried out using the PARIS Kit (Life Technologies, Shenzhen, Guangdong, China) based on the operating instructions.

### Cell Counting Kit Assays

Transfected Huh7 and HepG2 cells (4000 cells/well) were incubated at 37°C in an atmosphere with 5% CO_2_ for 24, 48, 72 and 96 h, respectively. CCK-8(Dojindo, Beiyu Biology, Nanjing, China) was used to incubate the above cells for 4 h. Using a microplate reader, the absorbance was observed at 450 nm.

### Colony Formation Assays

Cells (at a density of 3 × 10^3^ per well) were seeded in six-well plates. Subsequently, 4% paraformaldehyde (absin, Pudong, Shanghai) was applied to fix formed colonies for 30 min. 0.1% Crystal violet dye (Merck, Folaisi, Wuxi, China) was used to stain the cells for 15 min. Finally, our group counted and photographed the colonies.

### 5-Ethynyl-2’-Deoxyuridine Assay

EDU experiments were conducted for exploring cellular capabilities. Cells (6×10^3^) were placed in 48-well plates. Under the condition of 5% CO_2_ and 37°C, 50 μM of EdU diluent was used to cultivate cells for 2 hours. Then, PBS was used rinse the collect cells which was fixated by the use of paraformaldehyde. Next, Apollo 567 working solution was applied to process cells. For the counterstain of the nuclei, DAPI was added for 8 minutes. Finally, fluorescent microscopy was used to observe the collected cells.

### Scratch Test

Cellular scratch experiments were applied for the determination of the effects of LINC00882 on invasive abilities. Briefly, 1×10^6^ cells were inoculated into a 6-well plate and cultured for 24 h. After a pipette tip was applied to gently scratch the cells for the formation of the centers of the well, PBS was applied to wash the cells for 3 times. an inverted fluorescence microscope (Nikon, Tokyo, Japan) was applied for the observation of scratch distances.

### Tranwell Assays

The transwell assays were conducted to examine cellular invasion. Briefly, Huh7 and HepG2 cells were seeded on the top transwell chamber (60 µL of Matrigel at 1:7 dilution; Shenzhen, China) to the bottom chamber, 500 μL of medium was added. 90% ethanol was applied to fix the cells after 24 h. Besides, 0.5% crystal violet prior was applied to stain the collected cells. An inverted microscope was used for the observation.

### Dual-Luciferase Reporter Gene Assay

The wild-type and mutant LINC00882 containing miR-214-3p binding sites on the LINC00882 promoter region were ligated into the pMIR luciferase reporter vectors. Then, using Lipofectamine 2000 (Invitrogen, Xuanwu, Nanjing, China), we co-transfected the vectors into cells with LINC00882/NC mimic. To normalize the reporter luciferase activities, the dual luciferase reporter assays were carried out. In addition, with mutant-type and wild ATF2 binding sites, the LINC00882 promoter were inserted to pGL3 vector (Promega, Haidian, Beijing, China) for promoter assays.

### Chromatin Immunoprecipitation

The ChIP assays were performed following the methods and protocols descripted by the previous publications (20, 21). The HCC cells, HepG2, were transfected with vectors and harvested for the ChIP performed by using the ChIP kit (Promega Corporation, USA) according to the protocol by manufacture. About 2×10^9^ amount of HepG2 cells were fixed by 4% formaldehyde (v/v diluted by PBS) and the final concentration formaldehyde was 1%. Then, the mM dose of glycine solution was added and cells were cracked by the lysis-buffer. The nuclear sub-fraction of the cells was separated by centrifugation. Then, the nuclear sub-fraction lysates were sonicated to generate an average DNA fragments with the size of 0.5–1 kb. At last, the immunoprecipitation was performed with anti-Tubulin (α-, β- and γ- Tubulin) (Abcam Corporation, UK). The non-specific IgG was used as blank control. The DNA fragments in the complex (Tubulin with Centromere related sequence) was analyzed by the quantitative polymerase chain reaction. The DNA faction amplified was Human (hg38) chr2 q11.1: 92872758- 92873099. The primers are as followed: Forward sequence: 5’-tctgcaagaggatatttggatagc-3’; Reverse sequence: 5’-tcaccataggcctgaaagcg-3’.

### Western Blotting

Proteins were extracted from HCC cells, and then quantified. Subsequently, 30 μg of proteins was separated *via* 10% SDS-PAGE and transferred on nitrocellulose membranes (Pierce, Qingpu, Shanghai, China). After blocked with 3% BSA (YT0230-2, Yita Bio, Pinggu, Beijing, China), the membranes were incubated with primary antibodies against CENPM (ab243820, Abcam, Shenzhen, Guandong), and GAPDH (ab8245, Abcam, Shenzhen, Guandong) overnight at 4°C, and secondary antibody labeled with horseradish peroxidase (Abcam) for 2 h at room temperature. Subsequently, the BeyoECL Plus kit (LM0018, Lianmai Technology, Songjiang, Shanghai, China) was applied to develop the membranes. GAPDH was used to normalize the expressions of CENPM.

### Tumor Xenograft Analysis

The experimental procedures were approved by Animal Ethics Committee of National Cancer Center/National Clinical Research Center for Cancer/Cancer Hospital & Shenzhen Hospital. For *in vivo* assays, Vital River (Chaoyang, Beijing, China) provided 4-weeks old male BALB/c nude mice which were randomly divided into two groups: sh-LINC00882-1 group (n = 6) and sh-NC group (n = 6). LINC00882-1 or sh-NC-transfected Huh7 cells were subcutaneously injected into the right flank of nude mice. Digital calipers were applied to measure tumor volume every 4 days, and the formula (tumor volume = 1/2 (length × width^2^) was used to calculate volumes. 28 days later, the mice were sacrificed, followed by the examination of the weight of all tumors.

### Statistical Analysis

SPSS 19.0 software (SPSS Inc., Chicago, IL, USA) was used to analyze all data which are presented as means ± SD. Student’s t-test or one-way analysis of variance was applied to compare the differences between two groups or among multiple groups, followed by Tukey’s tests. A *p*<0.05 was considered statistically significant.

## Results

### Expressions of LINC00882 Are Increased in Human HCC Tissues

**Figure 1:**
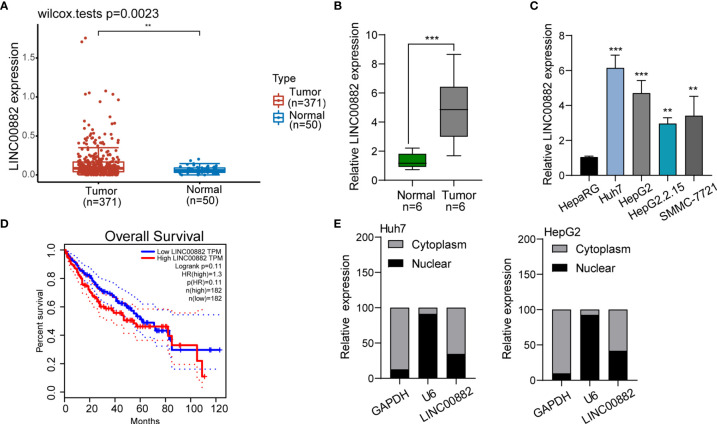
LINC00882 expression was increased in HCC. **(A)** Higher levels of LINC00882 were observed in HCC specimens (n=371) than normal liver specimens(n=50) from TCGA datasets. **(B)** The expression levels of LINC00882 in paired tissue samples from 6 patients were determined by RT-qPCR. **(C)** The level of LINC00882 in n HepaRG and four HCC cell lines. **(D)** Survival assays of 364 HCC patients from TCGA datasets. **(E)** Relative LINC00882 expression levels in nuclear and cytosolic fractions of Huh7 and HepG2 cells. **p < 0.01, ***p < 0.001.

To screen the possible lncRNAs involved in HCC progression, our group searched TCGA datasets and focused on LINC00882 which was distinctly overexpressed in HCC samples (n=371) compared with normal liver samples (n=50) (**Figure 1A**). The results of RT-PCR in samples of our patients also revealed that LINC00882 expressions in HCC tissues (n=6) were distinctly higher than those in the matched normal liver tissues (n=6) (**Figure 1B**). In addition, we observed that four HCC cells exhibited a higher level than HepaRG cells (**Figure 1C**). Survival assays based on TCGA datasets revealed that patients with higher LINC00882 expressions exhibited a poor trend of long-term survivals compared with those with lower LINC00882 expressions (**Figure 1D**). Given that lncRNA functions are dependent on its subcellular localization, we performed subcellular fractionation, finding that LINC00882 was mainly localized to the cytoplasm (**Figure 1E**).

### ATF2 Activates LINC00882 Transcription in HCC Cells

**Figure 2:**
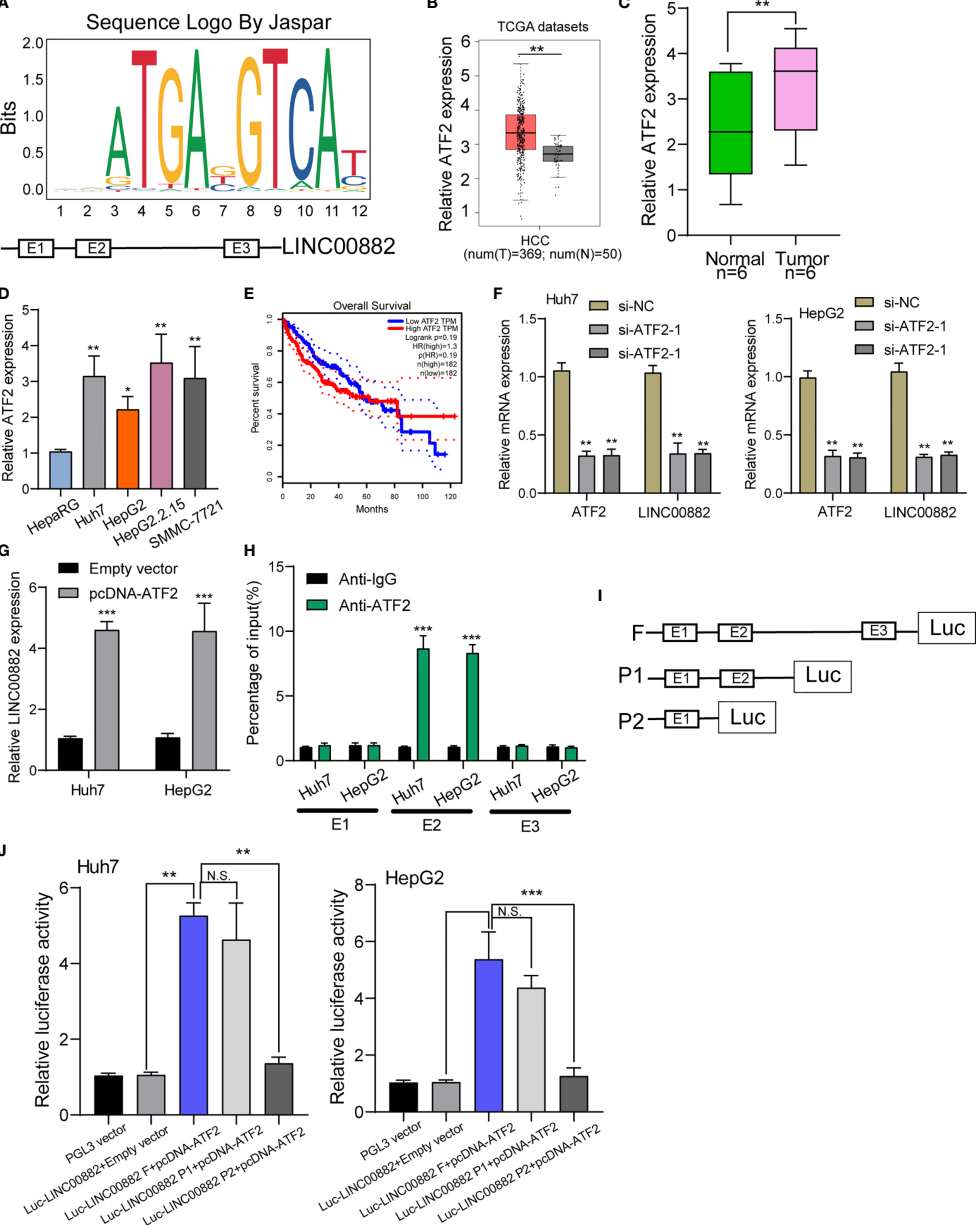
ATF2 Activates LINC00882 expression in HCC cells. **(A)** ATF2 binding site prediction in the LINC00882 promoter region using JASPAR. **(B)** By analyzing TCGA datasets, ATF2 expression was shown in HCC specimens (n=369) and normal liver specimens (n=50). **(C, D)** RT-PCR for ATF2 expression in HCC specimens from our patients and HCC cell lines. **(E)** Survival assays of 364 patients from TCGA datasets divided into two groups according to LINC00882 expression. **(F)** QRT-PCR for The levels of ATF2 and LINC00882 in HepG2 and Huh7 cells transfected with si-ATF2-1, si-ATF2-2 or si-NC. **(G)** Overexpression of ATF2 led to the promotion of LINC00882 levels in Huh7 and HepG2 cells. **(H)** ChIP-qPCR analysis of ATF2 occupancy in the LINC00882 promoter in Huh7 and HepG2 cells. **(I)** Construction of the luciferase reporter vector. **(J)** Dual luciferase reporter assays for the determination of the ATF2 binding site on the LINC00882 promoter region. *p < 0.05, **p < 0.01, ***p < 0.001, N.S., no significant.

Growing studies suggested that transcription factors such as E2F1, SP1 may exhibit regulatory effects on the expression of downstream targets including lncRNAs (22, 23). To predict the possible transcription factors, our group searched JASPAR database (http://jaspar.genereg.net/), finding that ATF2 may bound to the LINC00882 promoter region with strong probability (**Figure 2A**). Based TCGA datasets, ATF2 was highly expressed in HCC specimens, which was further confirmed in our cohort and four cell lines (**Figures 2B–D**). Survival assays with TCGA datasets revealed patients with higher ATF2 expressions showed a poor trend of overall survival in 40 months (**Figure 2E**). RT-PCR assays revealed that silence of ATF2 resulted in the obvious inhibition of LINC00882(**Figure 2F**), while its overexpression promoted the expression of LINC00882 (**Figure 2G**). By the use of ChIP assays, our group observed distinct ATF2-binding activities on the endogenous LINC00882 promoter region around E2 (**Figure 2H**). Besides, luciferase reporter assays demonstrated that ATF2 did not binds to the other two sites, but only the E2 binding site (**Figures 2I, J**).

### Silencing of LINC00882 Suppresses the Proliferation and Metastasis of HCC Cells

**Figure 3:**
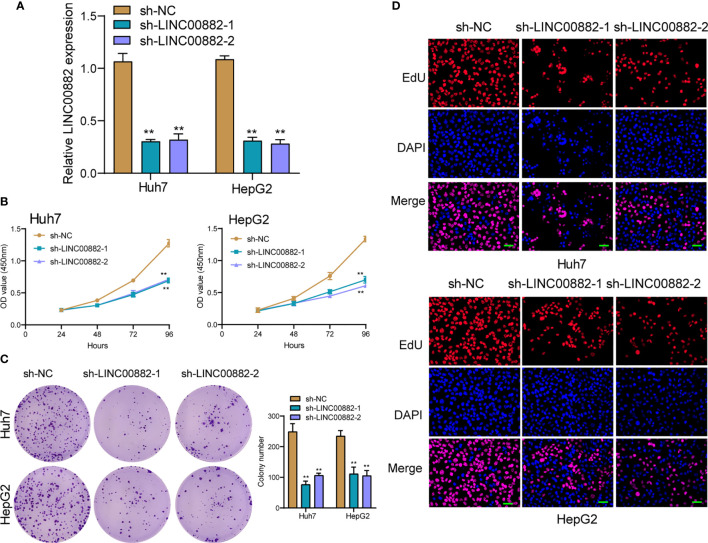
Knockdown of LINC00882 suppressed the proliferation of HepG2 and Huh7 cells. **(A)** The relative expressions of LINC00882 in Huh7 and HepG2 cells were distinctly reduced by sh-LINC00882-1 or sh-LINC00882-2 compared with the si-NC. **(B)** CCK-8 assays evaluated cell viability. **(C)** Clone number of Huh7 and HepG2 cell lines after transfected by sh-LINC00882-1, sh-LINC00882-2 or sh-NC. **(D)** Cell proliferation was determined by EdU assay in Huh7 and HepG2 cells. **p < 0.01.

**Figure 4:**
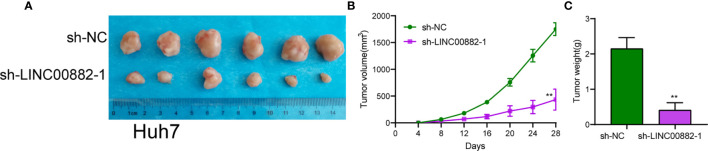
LINC00882 regulates HCC growth *in vivo*. **(A)** Tumors derived from mice in two different groups were presented. **(B, C)** Volume and weight of tumors obtained from two groups were measured and shown. **p < 0.01.

**Figure 5:**
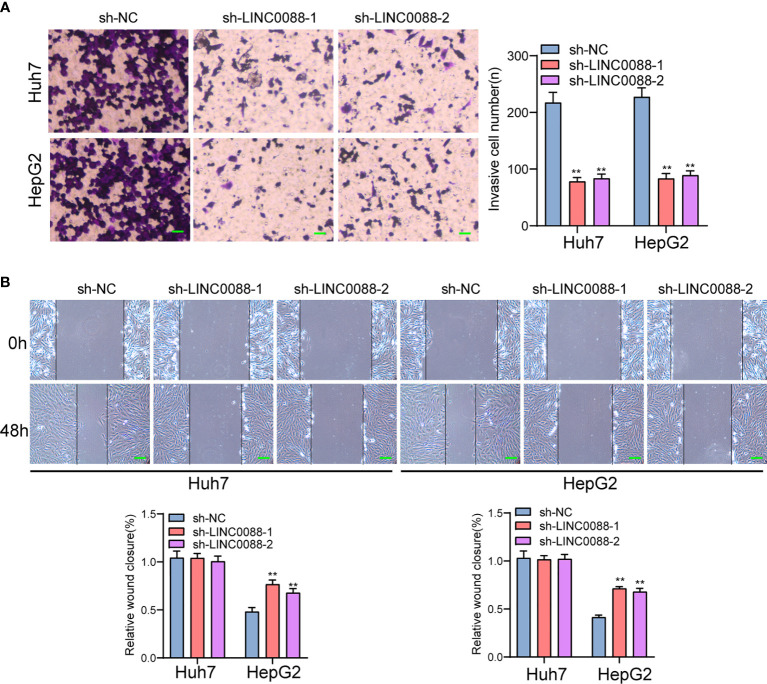
Knockdown of LINC00882 suppressed the migration and invasion of HCC cells. **(A)** Transwell experiments revealed knockdown of LINC00882 inhibited Huh7 and HepG2 cell invasion. **(B)** The wound healing assay showed a significant decrease of Huh7 and HepG2 cells migration after transfected sh-LINC00882-1 or sh-LINC00882-2. **p < 0.01.

To evaluate the roles of LINC00882 in HCC, Huh7 and HepG2 cells were transfected with shRNA (sh-LINC00882-1, sh-LINC00882-1) to silence the expression of LINC00882. As examined by RT-PCR, the expressions of LINC00882 were significantly inhibited by sh-LINC00882-1 and sh-LINC00882-2 in both HepG2 and Huh7 cells (**Figure 3A**). CCK-8 assays indicated that LINC00882 knockdown distinctly suppressed the proliferative capabilities of Huh7 and HepG2 cells (**Figure 3B**). Besides, the inhibitory effects of LINC00882 knockdown on HCC cell proliferation were also demonstrated by the colony formation and Edu assays (**Figures 3C, D**). For further exploration of the oncogenic functions of LINC00882, our group conducted *in vivo* experiments. As presented in **Figures 4A–C**, tumor volume and weight were reduced by sh-LINC00882-1 compared with scramble control. Furthermore, the results of Transwell assays and Wound-healing assays showed that the knockdown of LINC00882 weakened the invasion (**Figure 5A**) and migration (**Figure 5B**) ability of Huh7 and HepG2 cells. Therefore, LINC00882 was an oncogenic lncRNA in HCC.

### Reciprocal Modulation Between LINC00882 and miR-214-3p

**Figure 6:**
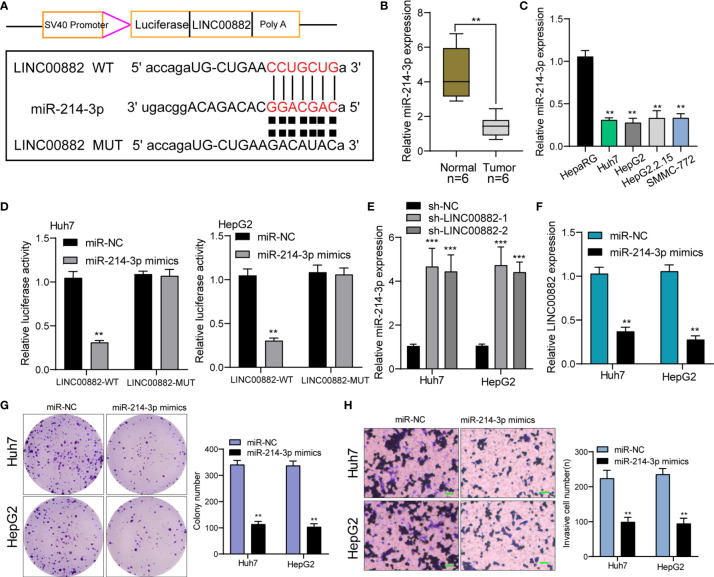
LINC00882 acts as a ceRNA for miR-214-3p. **(A)** LINC00882 containing the putative miR-214-3p recognition sites was cloned downstream of the luciferase gene. **(B, C)** RT-PCR for the LINC00882 expression in tumor specimens from our patients and cell lines. **(D)** Luciferase reporter assay was applied to verify the targeted binding effect between LINC00882 and miR-214-3p. **(E)** RT-PCR for miR-214-3p levels in Huh7 and HepG2 cells after LINC00882 knockdown. **(F)** The levels of LINC00882 levels in Huh7 and HepG2 cells after Overexpression of miR-214-3p. **(G)** Colony formation assay. **(H)** The invasive number of Huh7 and HepG2 after knockdown of LINC00882. **p < 0.01, ***p < 0.001.

We have confirmed that LINC00882 was mostly distributed in the cytoplasm, which suggested that LINC00882 might exert its biological function by sponging miRNA. Then, we searched Starbase which can predict the possible miRNAs binding sites for LINC00882. The binding sites of LINC00882 and miR-214-3p were presented in **Figure 6A**. RT-PCR assays indicated miRNA-214-3p was lowly expressed in HCC specimens and cell lines (**Figures 6B, C**). The luciferase activity of WT- LINC00882 instead of MUT-LINC00882 was distinctly reduced by miRNA-214-3p overexpression in Huh7 and HepG2 cells (**Figure 6D**). RT-PCR assays suggested miR-214-3p levels were distinctly upregulated in Huh7 and HepG2 cells after knockdown of LINC00882 (**Figure 6E**), while miR-214-3p overexpression distinctly suppressed the levels of LINC00882 (**Figure 6F**). Functionally, miR-214-3p upregulation was observed to inhibit the proliferation and invasion of HepG2 and Huh7 cells (**Figures 6G, H**).

### LINC00882 Directly Targets CENPM in HCC Cells

**Figure 7:**
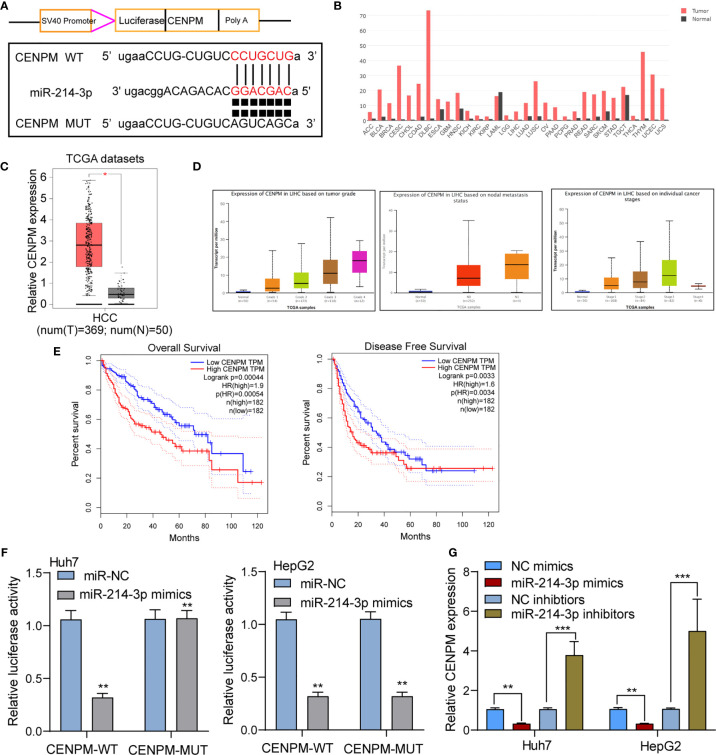
CENPM was identified as a direct target of miR-214-3p in HCC cells. **(A)** Bioinformatics tools reveal the complementary binding sites within miR-214-3p and CENPM. **(B)** Pan-cancer analysis of CENPM using TCGA datasets. **(C, D)** The expression pattern of CENPM in HCC specimens with different stages. **(E)** Survival assays of HCC patients according to the expression of CENPM by analyzing TCGA datasets. **(F)** Luciferase reporter assay validated the molecular binding. **(G)** RT-PCR determined the expression of CENPM in Huh7 and HepG2 cells transfected with NC mimics, miR-214-3p mimics, NC inhibitors or miR-214-3p inhibitors. *p < 0.05, **p < 0.01, ***p < 0.001.

To further study the mechanisms of actions of miR-214-3p in HCC, we performed bioinformatics assays to search the targets of miR-214-3p. CENPM was predicted to harbor putative binding sequences for miR-214-3p (**Figure 7A**). Then, we focused on CENPM due to its important functions during tumor progression (24, 25). Based on TCGA datasets, we observed CENPM displayed a high level in most types of cancers, and CENPM levels were distinctly upregulated in HCC specimens, especially in those with advanced stages (**Figures 7B–D**). Survival assays based on TCGA datasets revealed that high CENPM expressions were associated with poor clinical outcomes (**Figure 7E**). The results of luciferase reporter assay s showed that compared with CENPM 3’UTR-mut and miR-214-3p mimics, luciferase activity of Huh7 and HepG2 cells transfected with CENPM 3’UTR-wt and miR-214-3p mimics was distinctly decreased (**Figure 7F**). Moreover, overexpression of miRNA-214-3p distinctly suppressed the expressions of CENPM, while its silence displayed an opposite result(**Figure 7G**). On the other hand, we also performed *in vitro* assays to explore the function of CENPM in HCC. As shown in **Supplementary Figure S1A**, the results of RT-PCR and western blot showed that the expression of CENPM was distinctly decreased in Huh7 and HepG2 cells transfected with si-CENPM compared with si-NC. We further performed functional experiments, finding that knockdown of CENPM distinctly suppressed the proliferation (**Supplementary Figures S1B, C**), invasion (**Supplementary Figure S1D**) and migration(**Supplementary Figure S1E**) of Huh7 and HepG2 cells.

### LINC00882 Promoted HCC Progression by Decreasing CENPM Expression *via* Sponging miR-214-3p

**Figure 8:**
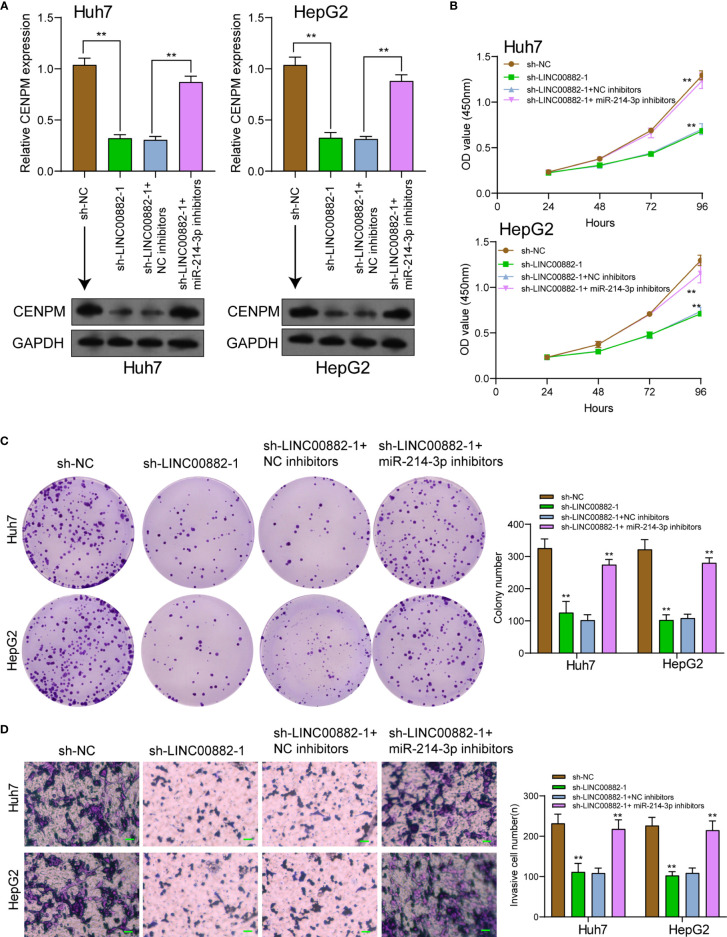
Knockdown of miR-214-3p effectively reversed LINC00882 knockdown-induced inhibition on HCC progression *in vitro*. **(A)** RT-PCR and Western blot examined the expression of CENPM in Huh7 and HepG2 cells transfected with sh-NC, sh-LINC00882-1, sh-LINC00882-1+ NC inhibitor or sh-LINC00882-1+ miR-214-3p inhibitor. CCK-8 assays **(B)**, colony formation assays **(C)** and Transwell assays **(D)** of HepG2 and Huh7 cells after transfection. **p < 0.01.

To demonstrate that LINC00882 aggravated the tumor behaviors of HCC through the modulation of miR-214-3p/CENPM axis, our group conducted rescue experiments *via* cotransfecting sh-LINC00882-1 or sh-NC and miR-214-3p inhibitors or NC inhibitors into HCC cells. As presented in **Figure 8A**, silence of miR-214-3p reversed the distinct suppression of LINC00882 silence on the expressions of CENPM. Besides, functional experiments revealed silence of miR-214-3p reversed the distinct inhibition of LINC00882 silence on the proliferative and invasive abilities of HCC cells (**Figures 8B–D**).

### ATF2/LNC000882/miR-214-3p Pathway Modulate The Binding of Tubulin With Centromere Related DNA Sequence by Targeting CENPM

**Figure 9:**
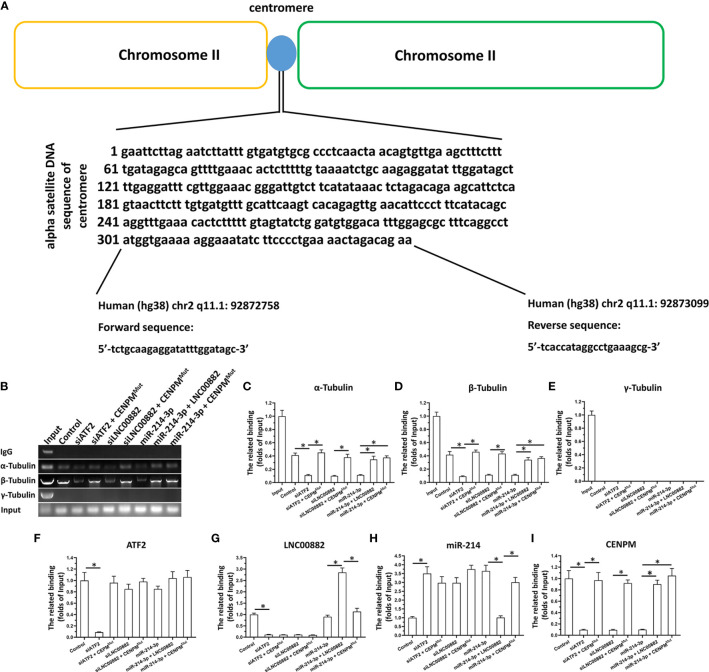
The effect of ATF2/LNC000882/miR-214-3p/CENPM pathway on the binding of Tubulin to Centromere related DNA sequence. **(A–E)** The HepG2 cells were transfected with vectors and harvested for the ChIP assays. The complex of Tubulin with Centromere related DNA sequence was separated by the antibodies of Tubulins. **(F)** The expression level of ATF2, **(G)** LNC000882, **(H)** miR-214-3p or **(I)** CENPM in HepG2 cells were examined by the qPCR. The results were shown as the schematic-diagram images of the Centromere related DNA sequence **(A)**, the images or mean ± SD from qPCR of the ChIP results **(B–E)** or the mean ± SD from the qPCR. *p < 0.05.

The chromatin immunoprecipitation (ChIP) was performed to detect the binding effect of Tubulin and Centromere related DNA sequence reflecting the influence of CENPM on the centromere-microtubule interaction to form the spindle. As shown in **Figures 9A–E**, as expected, α-Tubulin and β-Tubulin could binding to the Centromere related DNA sequence; however, the γ-Tubulin could not interacted with the Centromere related DNA sequence. Knockdown of ATF2, LNC000882 *via* siRNA or overexpression of miR-214-3p both resulted to the knockdown of CENPM and the decreased binding of α-Tubulin or β-Tubulin could to the Centromere related DNA sequence (**Figures 9F–I**). Rescued the expression of CENPM *via* transfection of CENPM with the mutated miR-214-3p targeting site in 3’UTR (CENPM^Mut^) could disrupt the effect of ATF2, LNC000882 knockdown or miR-214-3p overexpression. Moreover, the overexpression of LNC000882 also repressed the expression of miR-214-3p and enhanced the expression of CENPM. These results further confirmed the specificity of ATF2/LNC000882/miR-214-3p pathway modulate the binding of Tubulin with Centromere related DNA sequence by targeting CENPM.

## Discussion

Several studies reported the functions of lncRNAs in HCC progression (26). For instance, lncRNA-PDPK2P was shown to exhibit a higher level in HCC and accelerated invasion and tumor growth of HCC cells *via* PDK1/AKT/Caspase 3 pathway (16). LncRNA MCM3AP-AS1, a possible diagnostic factor for HCC, was reported to promote the growth of HCC through modulating miRNA-194-5p (27). The oncogenic or anti-oncogenic roles of lncRNAs encouraged us to further explore the important lncRNAs involved in HCC progression. Here, we identified a novel lncRNA-related oncogene in HCC. We provided evidences that LINC00882 levels were distinctly upregulated in HCC. Using TCGA datasets, we observed patients with high LINC00882 showed a poor trend in long-term survivals. Functionally, we demonstrated knockdown of LINC00882 distinctly inhibited the invasion, migration and proliferation of HepG2 and Huh7 cells, suggesting it as a tumor promotor in HCC progression. We searched literatures and only found LINC00882 was overexpressed in hepatocellular carcinoma and chromophobe renal cell carcinoma (18, 19). However, the studies involved in potential function of LINC00882 in tumors have not been found. Our findings may provide a new clue for the research of LINC00882 in other types of tumors.

Several studies have provided evidences that transcription factors can regulated the expressions of lncRNAs just like some protein-coding genes (28, 29). For instance, SP1-induced upregulation of lncRNA TINCR suppressed the metastasis ability of lung adenocarcinoma cells *via* regulating miR-107/RAB14 (30). Overexpressed lncRNA HAGLROS induced by STAT3 promoted the metastasis of gastric cancer (31). In HCC, several HCC-related lncRNAs, such as lncRNA ZFPM2-AS1 (by STAT1) and lncRNA RAET1K (by HIF1A), were also reported to be regulated by transcription factors (32, 33). In this study, we found ATF2 may bind directly to the LINC00882 promoter region, followed by a series of experiments confirming the overexpression of LINC00882 induced by ATF2. ATF2 is a member of the activator protein 1 (AP-1) TF family. Previously, several studies have reported ATF2 was highly expressed in several types of tumors and served as a tumor promotor, including HCC (34, 35). However, whether ATF2 can activate lncRNA transcription was rarely reported. Our finding firstly reported ATF2 overexpression resulted in the upregulation of LINC00882.

In recent years, a ceRNA mechanism was developed, which showed lncRNAs may serve as miRNA sponges, thereby competitively regulating the targets of miRNAs (36, 37). More and more evidences have shown that several functional lncRNAs could absorb miRNAs to regulate the expressions of anti-oncogenes or cancer promotors in tumor developments (38, 39). However, the ability of several lncRNAs with low abundance was not enough in sponging miRNAs. Many cellular evidences have demonstrated lncRNAs which exhibit high levels in the cytoplasm are ideal lncRNAs (40, 41). In this study, we observed that LINC00882 was mainly expressed in the cytoplasm, which provided a basic possibility for the ceRNA mechanisms. By the use of bioinformatics assays, miRNA-214-3p was predicted as a targeting miRNA of LINC00882, followed by the demonstration of luciferase reporter and RT-PCR. Previously, miR-214-3p was found to be lowly expressed in HCC and suppressed the proliferative and metastatic abilities of HCC cells, which was consistent with our findings (42, 43). On the other hand, we also found that miR-761 may be a potential target of LINC00882. However, miR-761 was found to act as a tumor promotor in HCC (44).

Centromere protein M (CENPM), encoding a kinetic protein, modulate chromosomal separation during cellular divisions (45). The levels of CENPM were observed to appear preferentially in immunity cells involving cancer specimens and cancer derived cells (24, 46). In HCC, CENPM silence resulted in the suppression of the proliferation and metastasis of HCC cells, and CENPM was regulated by several miRNAs (47, 48). It has been confirmed that miRNAs can target downstream genes and suppress their expression (49–51). Interestingly, using a combination of multiple biochemical analyses and mechanistic studies, CENPM was identified as a direct target of miR-214-3p. We also analyzed TCGA datasets, finding that CENPM was overexpressed in HCC. Knockdown of CENPM suppressed the proliferation and invasion of HCC cells. Finally, our group carried out rescue experiments, finding that silence of miR-214-3p reversed the distinct inhibition of LINC00882 knockdown on the expression of CENPM as well as the proliferation and invasion. Therefore, our study unveiled the contribution of the LINC00882/miR-214-3p/CENPM pathway in regulating HCC progression.

For the activity of CENPM itself, we have conducted various studies. Using siRNA to down-regulate the expression of CENPM can significantly inhibit the proliferation of HCC cells. At the same time, for the specific mechanism of CENPM itself, CENPM is a centromere protein, and its expression level is closely related to the normal function of the centromere-spindle. Because cellular immunofluorescence is difficult to quantitatively reflect the direct interaction between tubulin and centromere, we use chromatin immunoprecipitation technology to detect the combination of tubulin and centromere DNA sequence, and finally perform specific effects on CENPM Sexual analysis. Our results show that down-regulating the expression of CENPM can inhibit the binding of tubulin α and β to the centromere sequence. At the same time, the specificity of the action of ATF2/LNC000882/miR-214-3p is determined by setting a reasonable control. Moreover, Tubulin is mainly composed of α- and β-subtypes, while γ-subtypes are mainly distributed in the centrosome. The results of this study also confirmed that in the ChIP experiment, only α-Tubulin and β-Tubulin can interact with centromere DNA sequences, while γ-Tubulin cannot interact with centromere DNA sequences. These methods and results have advantages and innovations: the combination of centromere and tubulin is the basis for the formation of spindles and cell proliferation. The use of chromatin immunoprecipitation technology to detect the combination of tubulin and centromere can be quantitative and intuitive Reflects the interaction between microtubules and centromeres, which is an ideal method for this type of research.

However, this study has several limitations. Firstly, the sample size was relatively small, we will collect more samples for research in the future. Secondly, additional mechanisms of LINC00882 such as methylation in regulating HCC progression require further study.

## Conclusion

Based on results, LINC00882 may be a tumor promotor for HCC, and ATF2 could activate its transcription. LINC00882 exerted oncogenic roles in modulating the proliferation and metastasis of HCC cells. Our findings firstly suggested the underlying mechanisms behind this development, that LINC00882 drove HCC progression by increasing CENPM levels through sponging miR-214-3p, providing a novel therapeutic target for HCC treatments.

## Data Availability Statement

The raw data supporting the conclusions of this article will be made available by the authors, without undue reservation.

## Ethics Statement

The studies involving human participants were reviewed and approved by National Cancer Center/National Clinical Research Center for Cancer/Cancer Hospital & Shenzhen Hospital, Chinese Academy of Medical Sciences and Peking Union Medical College. The patients/participants provided their written informed consent to participate in this study. The animal study was reviewed and approved by National Cancer Center/National Clinical Research Center for Cancer/Cancer Hospital & Shenzhen Hospital, Chinese Academy of Medical Sciences and Peking Union Medical College.

## Author Contributions

HR, Z-cW, and XC conceived and designed the experiment. HR, Z-cW and Y-xS performed the experiments and wrote the manuscript. C-yQ and W-jZ performed the statistical analyses and generated the figures. HR, WZ, and TL collected the public data. Z-cW and XC collected the patient samples. HR and XC revised the manuscript. All authors contributed to the article and approved the submitted version.

## Funding

This study was supported by the Sanming Project of Medicine in Shenzhen(No.SZSM201612063 & No.SZSM201911008) and the Shenzhen Basic Research Program(KJYY20170412153658082).

## Conflict of Interest

The authors declare that the research was conducted in the absence of any commercial or financial relationships that could be construed as a potential conflict of interest.

## Publisher’s Note

All claims expressed in this article are solely those of the authors and do not necessarily represent those of their affiliated organizations, or those of the publisher, the editors and the reviewers. Any product that may be evaluated in this article, or claim that may be made by its manufacturer, is not guaranteed or endorsed by the publisher.
