# Supplementary figures and images for: ATF2-Induced Overexpression of lncRNA LINC00882, as a Novel Therapeutic Target, Accelerates Hepatocellular Carcinoma Progression via Sponging miR-214-3p to Upregulate CENPM
Source: Front Oncol. 2021 Aug 27;11:714264. doi: 10.3389/fonc.2021.714264 (PMC8429907; doi:10.3389/fonc.2021.714264)

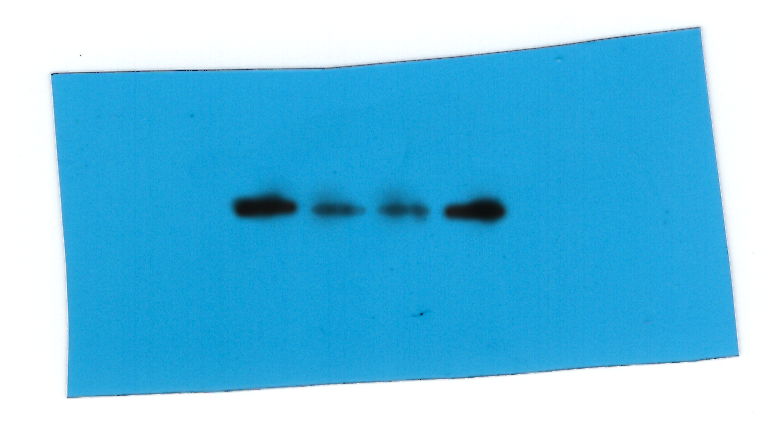

Supplement: Supplementary file 1 [file DataSheet_1.zip › Orignal Western Blots/CENPM-HepG2-Figure 8A.tif]

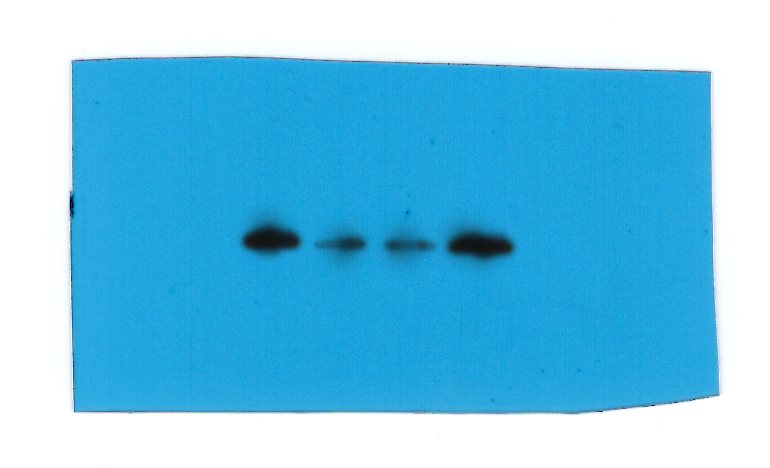

Supplement: Supplementary file 1 [file DataSheet_1.zip › Orignal Western Blots/CENPM-Huh7-Figure 8A.tif]

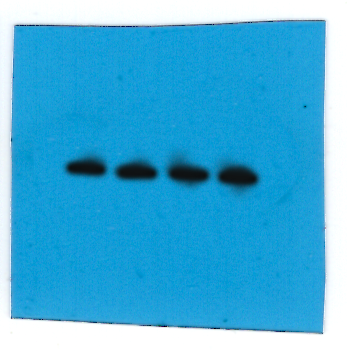

Supplement: Supplementary file 1 [file DataSheet_1.zip › Orignal Western Blots/GAPDH-HepG2-Figure 8A.tif]

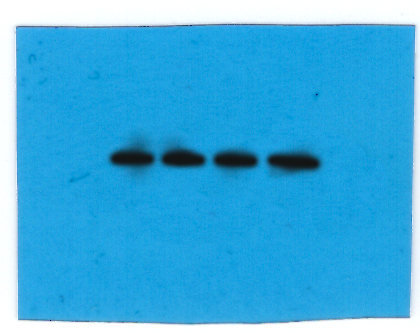

Supplement: Supplementary file 1 [file DataSheet_1.zip › Orignal Western Blots/GAPDH-Huh7-Figure 8A.tif]
