# Supplementary material for: ATF2-Induced Overexpression of lncRNA LINC00882, as a Novel Therapeutic Target, Accelerates Hepatocellular Carcinoma Progression via Sponging miR-214-3p to Upregulate CENPM
Source: Front Oncol. 2021 Aug 27;11:714264. doi: 10.3389/fonc.2021.714264 (PMC8429907; doi:10.3389/fonc.2021.714264)
Supplement: Supplementary file 2 [file DataSheet_2.docx]

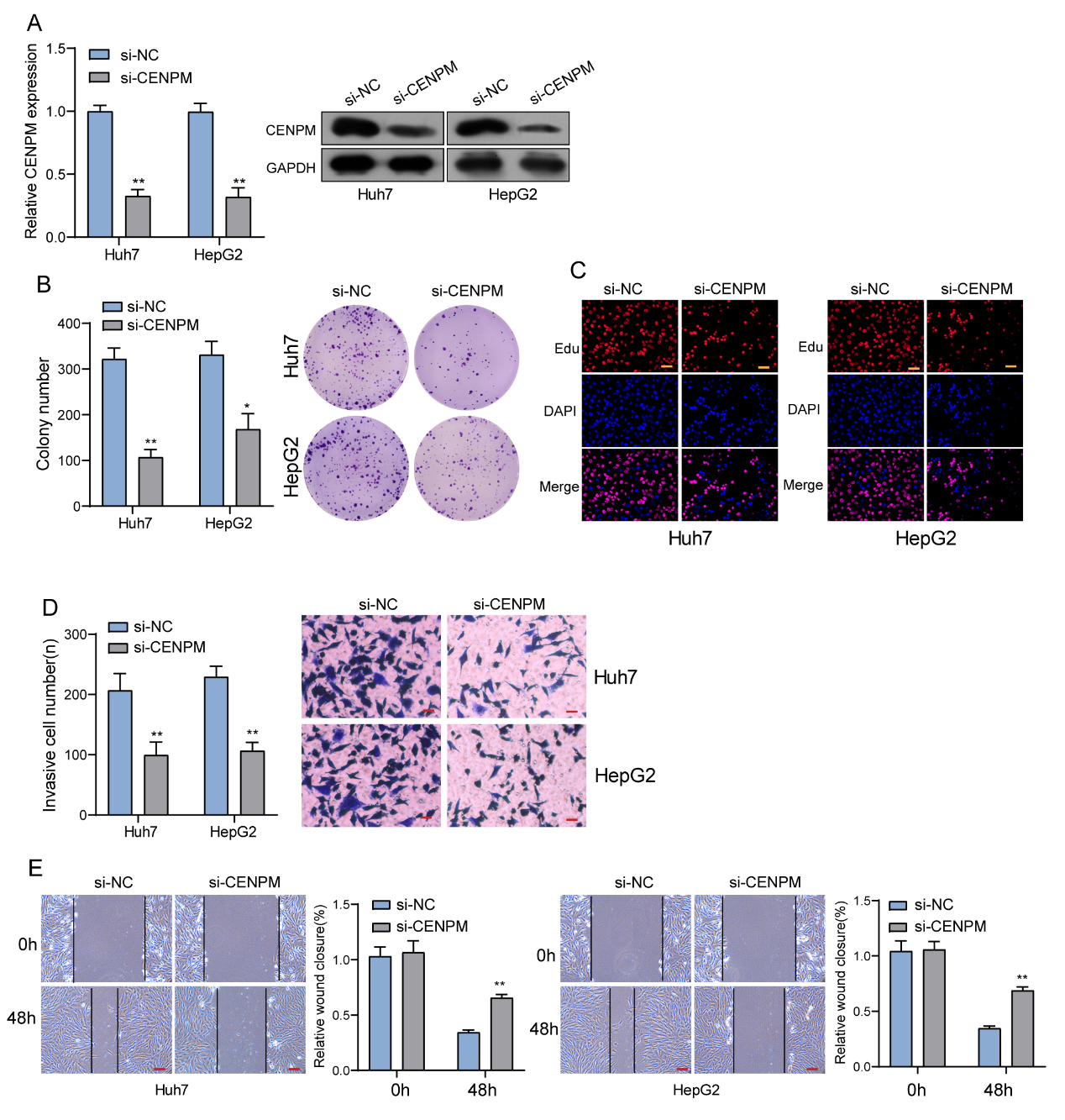


**Supplement Figure S1** The function of CENPM silence in HCC progression. (A) RT-PCR and Western blot confirmed the transfection efficiency of si-CENPM. (B) Colony formation assays, (C) Edu assays, (D) Transwell assays (E) The wound healing assays were performed to explore the effects of CENPM knockdown on HCC cells. *p<0.05, **p<0.01.
